# Community-based participatory interventions to improve food security: A systematic review

**DOI:** 10.3389/fnut.2022.1028394

**Published:** 2022-12-19

**Authors:** Azam Doustmohammadian, Fatemeh Mohammadi-Nasrabadi, Nastaran Keshavarz-Mohammadi, Melika Hajjar, Sepideh Alibeyk, Maryam Hajigholam-Saryazdi

**Affiliations:** ^1^Gastrointestinal and Liver Diseases Research Center, Iran University of Medical Sciences, Tehran, Iran; ^2^Research Department of Food and Nutrition Policy and Planning, Faculty of Nutrition Sciences and Food Technology, National Nutrition and Food Technology Research Institute, Shahid Beheshti University of Medical Sciences, Tehran, Iran; ^3^School of Public Health and Safety, Shahid Beheshti University of Medical Sciences, Tehran, Iran; ^4^Department of Community Nutrition, School of Nutrition Sciences and Food Technology, Student Research Committee, National Nutrition and Food Technology Research Institute, Shahid Beheshti University of Medical Sciences, Tehran, Iran; ^5^Faculty of Nutrition Sciences and Food Technology, Library, National Nutrition and Food Technology Research Institute, Shahid Beheshti University of Medical Sciences, Tehran, Iran

**Keywords:** food security, community-based participatory research, intervention, systematic review, food insecurity

## Abstract

**Introduction:**

This systematic review aimed to evaluate community-based participatory (CBP) interventions to improve food security and/or its dimensions to highlight the scope and characteristics of interventions and extract the characteristics of effective interventions.

**Methods:**

The electronic databases, including PubMed/MEDLINE, SCOPUS, EMBASE, Web of Science, and Google Scholar, were searched from 1980 to 30 August 2022 for relevant studies. We included randomized controlled trials (RCTs), cluster randomized controlled trials (cRCTs), controlled before and after studies (CBAs), non-randomized controlled trials (nRCT), and interrupted time series (ITS) studies to identify the community-based participatory interventions. The indicators of food and nutrition security into four dimensions, as well as food insecurity measured as score and/or prevalence of food insecurity based on validated perception-based measures were considered outcome. Two reviewers independently evaluated the studies for eligibility, extracted data, and evaluated the risk of bias in the included studies using the Effective Public Health Practice Project (EPHPP). The quality of included reports was categorized as strong (when there were no weak ratings), moderate (when one factor was rated as weak), or weak (when two or more factors were rated as weak). A descriptive analysis of the findings was performed.

**Results:**

A total of twelve studies were included. The quality of all eligible studies (*n* = 12) was rated as moderate/weak. Most CBP interventions were guided by formative research (*n* = 9, 75%). Two main groups for utilized strategies were identified: agricultural and nutrition strategies. Agricultural strategies included agricultural education, preparing and improving soil and seeds, promoting and supporting gardening/harvesting utilizing traditional skills based on the local culture, and agroecological practices. Nutrition strategies included store and shopping programs, farmers’ markets, fresh fruit and vegetable programs, nutrition education programs for mothers, and food vouchers. The main outcomes improved in the CBP interventions were food security (*n* = 2) and its dimensions, including availability (*n* = 3), access (*n* = 5), and utilization (*n* = 2). All agroecological practices achieved statistically significant outcomes in the intended food security target(s). However, nutritional interventions were not effective for some access components such as mean adequacy ratio, fruit and vegetable intake, and nutrition environment of the stores. No studies evaluated stability outcome components of food security.

**Discussion:**

CBP interventions guided by formative research data and agroecological practices were promising strategies to improve food security and its dimensions. Insufficient data on the stability components of food security and weak design studies were the considerable gaps in the research evidence reviewed. More research employing randomized experimental designs with adequate sample size and high retention rates is required.

**Systematic review registration:**

[https://www.crd.york.ac.uk/prospero/], identifier [CRD42020189477].

## Introduction

Food insecurity is a key challenge in fighting hunger and malnutrition and achieving health, as reflected in Sustainable Development Goals SDG1, SDG2, and SDG3 (1). Food security as a multi-dimensional phenomenon exists when all people, at all times, have social, physical, and economic access to safe, sufficient, and healthy food that meets their food preferences and dietary needs for a functional and healthy life (2). This definition identifies four essential dimensions of food security: “physical availability, economic and physical access to food, food utilization, and stability of the other three dimensions over time” (3). Food security can be measured at household, community, and national levels. At the national level, the focus is on hunger and poverty as the result of food consumption that is continuously insufficient to meet dietary energy requirements. Measurement is typically indirect and based on Food Balance Sheets (FBS), national income distribution, and consumer expenditure data. Direct experiential perception-based questionnaires and diet quality assessments based on food intakes are also used for measuring food security at household or individual levels (4). Global studies show an increase in food insecurity worldwide, and so its side effects include micronutrient deficiency, particularly in Asia, Latin America, and Africa (5). The global assessment of food security and nutrition report (6) indicated that 9.2 percent of the world’s population (about 700 million people) had possibly experienced hunger, a severe level of food insecurity. Adding a moderate level of food insecurity, the projected total of 26.4 percent of the world population (2 billion people) did not enjoy food security (6). Many efforts and interventions have been conducted worldwide to improve food insecurity by utilizing diverse strategies (7–10). Reviewing this intervention research allows lessons for future effective programs tackling hunger and food insecurity.

Strengthening community action is one of the five key health promotion actions recommended by the Ottawa charter for health promotion (11) and is still acknowledged as an essential health promotion strategy (12). Community-based participatory (CBP) intervention theory suggests that engaging community members as collaborators in the interventions to reduce health disparities is powerful on multiple levels (13). Developing a research project from the bottom-up (identification of critical issues to a particular population by community members) rather than the traditional top-down approach (identification of an agenda by researchers which may not reflect the community’s needs) would more likely improve a population’s participation and enthusiasm for the project and its intervention (14).

The community-based participatory approach highlights nurturing, deploying, and sustaining community partnerships that share leadership in planning, implementing, and evaluating evidence-based, creative, and culturally sensitive interventions that improve the application of research outcomes for community development and policy change (14, 15). CBPR requires the purposeful engagement of the community and stakeholder groups, taking advantage of their exceptional assets and prospects. Among the rewards of CBPR are university-community engagement, improved relevance and so the effectiveness of research, improved participant recruitment, and improved participatory research capacities among communities, as well as researchers and research and academic organizations, which changes the long-standing unequal power dynamics among them (16–20).

Hence, community-based participatory intervention is considered a valuable strategy to motivate community action, boost community development, and enhance programs’ sustainability and impact. However, there are challenges in implementing community-based participatory interventions. For example, they are time-consuming, difficult to implement, and difficult to convince academics and funding authorities (12, 21). Nonetheless, several studies have shown CBPR to be a successful method for addressing health outcomes (22–26). Reviewing the available studies (27–29) shows insufficient evidence to recommend a specific type of CBPR intervention or research to improve food security.

This systematic review aimed to identify, evaluate, and synthesize research results to create a summary of current evidence for effective community-based participatory food security interventions. Hence, it can contribute to preventing or reducing implementation mistakes, wasting time and resources, especially in developing countries/low-income communities that suffer from the shortage of resources, and maximize the cost-effectiveness of food security interventions. It may contribute to identifying research gaps at the global level.

More specifically, the review aimed to address these questions:

• What were existing interventions on food security and/or its dimensions developed using community-based participatory research (CBPR)?
• What are the characteristics of community-based participatory food security interventions?
• What are the characteristics of effective interventions aiming to improve food security and/or its dimensions (availability, access, utilization, and stability)?

## Methods

The current systematic review was undertaken based on the Preferred Reporting Items for Systematic review and Meta-Analysis (PRISMA) guideline (30). The protocol was registered in the PROSPERO under registration number CRD42020189477.

The Ethical Committee of Iran University of Medical Sciences (IR.IUMS.REC.1399.973) approved the study^1^, and its protocol was registered in the PROSPERO under registration number CRD42020189477.

### Data sources and search strategy

The primary literature source was a structured search of major electronic databases, from 1980 to 30 August 2022, including PubMed/MEDLINE, SCOPUS, EMBASE, and Web of Science. We chose 1980 as the starting point because this is when the term “food security” which includes food availability, access, utilization, and stability, began to be used (31). For finding gray literature, Google Scholar was searched up to page 20 (first 200 results) for title searches using the following keywords and was performed in duplicate: [(food insecurity) or (food security) or (food availability) or (food accessibility) or (food supply) or utilization or stability] AND [(community-based participatory) or CBP]. Databases were searched using a combination of free-text and medical subject heading (MeSH) search terms, text words, and keywords based on each database attribute, focusing on food security and its indicators of MeSH synonyms. Search strategies based on the PICO format (Participant, Intervention, Comparison, and Outcome) (32) and the MeSH database are presented in Supplementary Table 1.

A manual search of reference lists of included studies, related reviews, and documents was conducted to identify other relevant studies.

### Eligibility criteria and study selection

This review summarized only the evidence of quantitative studies. Therefore, we excluded qualitative studies during the selection process. Studies were included in the review if they measured primary outcomes quantitatively based on the food security framework (33). We excluded non-English articles, reviews, methodological articles, and conceptual papers (e.g., non-experimental studies). Studies with the following designs were included: randomized controlled trials (RCTs), cluster randomized controlled trials (cRCTs), controlled before and after studies (CBAs), and non-randomized controlled trials (nRCTs). As the main focus of this study was to identify community-based interventions, occupational and clinical studies were excluded from the review.

We examined all sex and age groups and socio-economically disadvantaged groups in developed and developing countries. Countries were grouped based on the economic classification of the World Bank^2^.

The study eligibility and exclusion criteria are presented in Table 1.

**TABLE 1:** Study eligibility and exclusion criteria based on the PICOS elements.

Inclusion criteria	
Participant	All sex and age groups and socio-economically disadvantaged groups in developed and developing countries.
Intervention	Community-based participatory (CBP) interventions to improve food security and its dimensions, including (34–38). **1) Food availability, through:** ● Infrastructure development (e.g., wastage control, marketing strategies). ● Agriculture and food security programs (e.g., monetary support for farmers, land assignment-security. ● Food security capacity-building in agriculture and/or other food production). ● Local vegetable gardening. ● Policies and trade regulations. **2) Food accessibility, through:** ● Income-generation cash transfer schemes and opportunities to improve buying power; ● Policies, vouchers, discounts, and subsidies addressing food prices; ● Social environment and social support interventions, including social support from family, neighbors, or government. **Food utilization, through:** ● Food literacy improvement regarding knowledge empowerment and skills building (e.g., Nutrition Education and Skills Training (NEST) program (37), interventions related to healthy food selections, cultural aspects that influence food utilization, choice, and allocation within the household). ● Knowledge and skill-based education about food safety. **3) Food stability, through:** ● Improved production and productivity of agriculture in a sustainable method, including more comprehensive, more equitable access to inputs (e.g., seed, water, fertilizers, and credit) by smallholder farmers, including women farmers (73)
Comparison	All comparisons, including different educational interventions; various strategies of delivery, educational information, intervention dosages, or the like; ordinary care; with or without control groups.
Outcome	Outcome measures considered as the indicators of food and nutrition security dimensions were presented in Table 2.
Study design	Cluster randomized controlled trials (cRCTs), randomized controlled trials (RCTs), non-randomized controlled trials (nRCT), controlled before and after studies, time series (ITS) studies.
Setting	Schools, homes, worksites, churches, and community (individual/household level).
Approach	Community-based participatory research approach includes the engagement of stakeholders in the following: ● Monetary responsibility for grant funds; ● Research method; ● Building collaboration; ● Preparation of measurement tools and data collection; ● Development and performance of interventions; ● Interpretation, dissemination, and applying the result.
Exclusion criteria	Irrelevant study design, including reviews, qualitative studies, conceptual documents and methodological articles, and ● Irrelevant participant(s), including participants with specific diseases or conditions); ● Irrelevant setting(s) (e.g., clinics and hospitals); ● Irrelevant intervention(s), including interventions that addressed transient food insecurity (e.g., food assistance during wars and natural disasters); ● Irrelevant outcome(s); ● Publications not English.

PICOS, Population, Intervention, Comparison, Outcome, Study design/Setting (32).

Food security interventions utilizing a community-based participatory (CBP) approach in the execution of research were included in the review. The CBP approach requires stakeholders’ engagement in selecting a research question, financial responsibility for grant funds, study design, building partnerships, developing measurement tools, collecting data, developing and implementing interventions, interpreting and disseminating, and applying the results (14, 23). To assist in identifying food security interventions, drawing from current evidence and framework, we defined a set of indicators for food security and/or its dimensions as the outcome measure of interest (34–38) (Table 2). Outcome measures were considered the indicators of food and nutrition security into four dimensions as well as food insecurity measured as score and/or prevalence of food insecurity based on validated perception-based measures.

**TABLE 2:** Indicators of food security as primary outcomes of community-based participatory interventions to improve food security in developing countries.

Measurement approach	Dimensions	Food security indicators
Direct	**−**	● Food insecurity
Indirect	Availability	● Average dietary energy supply adequacy; ● Average food production value; ● Dietary energy source derived from roots, cereals, and tubers; ● Average protein source; ● Average source of animal protein.
	Access	● Index of domestic food price index; ● Gross domestic product per capita (in purchasing power equivalent); ● Undernourishment prevalence; ● Food inadequacy Prevalence; ● Food deficit Depth; ● Share of food expenses of the poor.
	Utilization	● Percentage of wasting under-five children; ● Percentage of stunted under-five children; ● Percentage of underweight under-five children; ● Percentage of underweight adults; ● Anemia prevalence among pregnant women; ● Anemia prevalence among under-five children; ● Vitamin A deficiency Prevalence (forthcoming); ● Iodine deficiency prevalence (forthcoming).
	Stability	● Import-to-export ratio of foodstuffs; ● Non-violence/terrorism and political stability; ● Domestic food price volatility; ● Cereal import dependency ratio; ● Per capita food supply variability; ● Per capita food production variability.

Adapted from FAO, IFAD, and WFP (71).

Interventions involved participants drawn from the target population in community settings such as schools, churches, and workplaces, as well as interventions that involved the community in research (39).

Study eligibility and exclusion criteria based on the PICOS elements are presented in Table 1.

**FIGURE 1:**
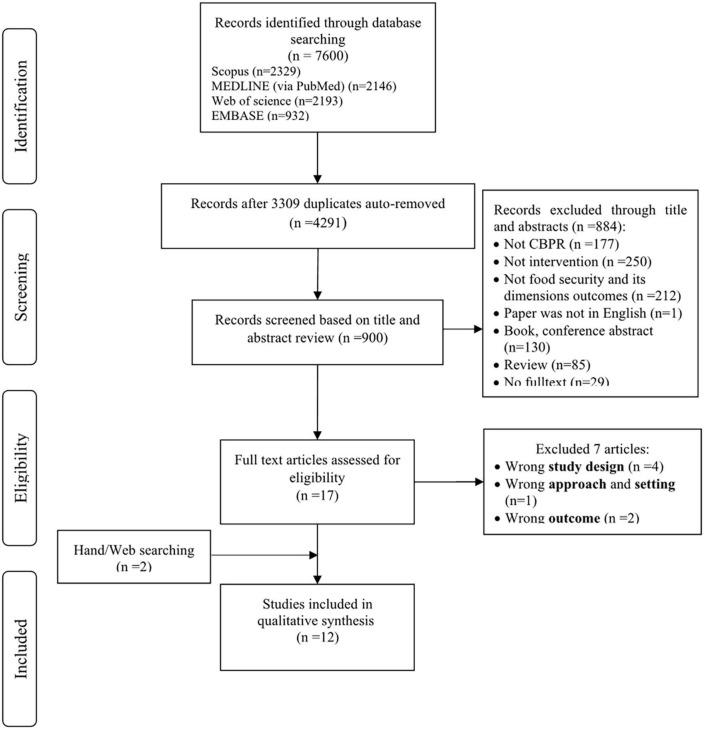
PRISMA flow diagram of the included studies. RCT, randomized control trial.

We transferred all the electronic search results into Endnote, and de-duplication was systematically done through The Systematic Review Assistant-De duplication Module (SRA-DM) (40). Two reviewers (AD and FMN) independently evaluated the remaining studies for eligibility. Any conflicts were resolved by a third independent reviewer (NKM) or by discussion. We carried out a full-text screening. Exclusion reasons for any excluded study were documented. We provided A PRISMA flow chart detailing the number of screened studies and included them in the review with exclusion reasons at each stage (Figure 1). A table of reasons for the studies’ exclusions was also designed (Supplementary Table 2).

### Data extraction and management

A standardized data extraction form was developed, included the study characteristics (first author, publication year), study methods (study design, unit of allocation or exposure, study period, and setting), participants (sample size, age, and ethnicity), intervention (description, intervention objectives, content, provider characteristics, control, and intervention groups) and outcomes of interest (change in food security status and/or its indicators including availability, access, utilization, and stability resulted from the community-based participatory interventions). Two authors (AD and MH) independently extracted data based on the pilot-tested inclusion criteria checklist. We contacted the authors by email to obtain the original report to see if there was any missing information or unclear data in the primary articles and reports.

### Methodological quality assessment

Selected papers were assessed by two reviewers using the validated Quality Assessment Tool for Quantitative Studies. This tool was constructed by the Effective Public Health Practice Project (EPHPP) (41) to assess the quality of included studies in systematic reviews relating to public health topics (42). Seven elements of the quality assessment tool were included: selection bias, study design, blinding, confounders, withdrawals/dropouts, and data collection methods, which resulted in an overall rating of strong, moderate, or weak (42): (a) strong (when there were no weak rating); (b) moderate (when one factor was rated as weak); and (c) weak (when two or more factors were rated as weak).

The quality assessment of all the included studies was conducted by two authors (AD and MH), and potential conflicts were resolved through discussion.

## Results

### Study selection

The initial search yielded 7600 potentially relevant studies (Scopus = 2329, PubMed = 2146, Web of science = 2193, and EMBASE = 932). After removing 3309 duplicates, we screened the remaining 900 studies based on the title and abstract review. The first stage selection excluded 884 studies based on the predefined exclusion criteria. Studies were mainly excluded as they were not interventional, CBPR, or did not evaluate food security and its dimensions outcomes, etc. Of these, 16 articles were potentially eligible for full-text reviewing. The full texts were retrieved for further assessment, with seven failing to meet the inclusion criteria. The main reason for excluding full texts was the wrong study design. Finally, a total of twelve studies were included in the final review.

### Characteristics of the studies

Of the twelve included articles in the review, six (50%) were agricultural interventions which all were conducted in developing countries, and six (50%) were nutritional interventions which most of them (83.3%) were conducted in developed countries (poverty regions).

Most of the studies were nRCT studies (*n* = 10), including pre-post intervention studies (*n* = 6) (43–48), cross-sectional studies with two groups’ comparisons (*n* = 3) (49–51), and longitudinal pre-post and delayed intervention (*n* = 1) (52). Two included studies were RCTs (41, 53). The duration of the intervention varied from four to 72 months across studies (Table 3).

**TABLE 3:** Baseline characteristic of included studies (*n* = 12).

	No.	First Author, year	Country	Study design	Target population (n, age, ethnicity)	Setting (level)	Intervention description (components, strategies, and study groups)	Duration	Outcomes measure (scale/methods)	Key findings
**Agricultural interventions/Developing countries**										
Non-randomized controlled trials	1.	Boedecker et al. (51)	Western Kenya	Cross-sectional study (two groups comparisons)	444, Children 12–23 months, women 15–49 years	Community (household)	● Workshops encourage and support communities in planning agricultural activities to improve nutrition, raising awareness on nutrition and healthy diets, identifying poultry raising, and kitchen gardening (especially traditional legumes and leafy vegetables) to support diet diversification. ● The workshops were led by a nutritionist of the country’s Ministry of Health (MoH) to share nutrition contents, Bioversity International and CHVs [I: 5 sublocation (*n* = 296) and C: 5 sublocation (*n* = 148)]	12 months	Mean dietary diversity score/DDS, the percent of women and children reaching minimum dietary diversity/MDD, and micronutrient adequacy using mean adequacy ratio/MAR	The intervention significantly effect on children’s mean DDS (the size of treatment effect = 0.7, *p* < 0.001) and children reaching MDD (the size of treatment effect = 0.2, *p* < 0.001)
	2.	Carney et al. (43)	Oregon	Intervention study (Pre-post survey)	131, 4–44 years	Community (household)	● The strategies provided by the community meeting included the project materials, such as seeds, and gardening strategies, such as preparing the land, choosing plants, compost, organic approaches for pest control, maintaining the garden, and harvesting the vegetables	43 months	Food security (accessibility), vegetable intake, food safety	In adults, vegetable intake increased from 18.2 to 84.8% (*p* < 0.001). Children’s vegetable consumption increased from 24.0 to 64.0% (*p* = 0.003).
	3.	Kansanga et al. (50)	Malawi	Cross-sectional study (two groups comparisons)	914, ≥30 years	Community (household, farmers)	● Using agroecological practices improves farmers’ knowledge, agricultural productivity, and household nutrition. In order to improve agricultural productivity and household nutrition, the project trained smallholder farmers on applying agroecological practices using local resources and a farmer-to-farmer knowledge-sharing approach. (I: agroecology-practicing farming households (*n* = 514). C: non-agroecology households (*n* = 400)	60 months	household production diversity and dietary diversity	A significant positive effect was shown in the mean dietary diversity (β = 0.175, *p* < 0.01) and production diversity of households (β = 0.289, *p* < 0.01).
	4.	Kazige et al. (48)	Mushing area, Walungu territory, South-Kivu province, eastern DRC (Congo)	Intervention study (Pre-post survey)	Rural household, NR	Community (household)	● In this study, to improve the food security of rural households in South Kivu, residues of four staple crops, including banana, cassava, maize, and common bean, were valorized in two stages: (1) monitoring of the fields of farmers throughout the cropping season to record the weight of crop residues and yields, and (2) evaluation of the potential of staple crop residues for mushroom production. A total of 70 fields were selected for this purpose	10 months	Effects of substrate ingredients (common bean, banana, cassava, and maize) and the additive used to increase production	43.5% of the households consumed common beans as vegetables, and 20.7% sold them at the local market. Maize gave the highest mushroom yields (2.4 kg kg^–1^)
	5.	Madsen (52)	Malawi	Longitudinal pre-post and delayed intervention	537, 43 years,	Community (farmers)	● Training on soil management, ● Soil compost/manure application, mulching, crop rotation, and agroforestry. Acquaintance of the participants about recipes with new products and the re-introduction of grains and legume crops [I: received intervention from the beginning (*n* = 428) and C: delayed intervention (*n* = 109)]	36 months	Food security ● Household Food Insecurity Access Scale-HFIAS (HFIAS)	Crop yield diversity increased in intervention farms from a mean of 2.06 crops to 4.23 crops. The percentage of food security increased from 10% at the baseline to 30% at the end line.
	6.	Nyantakyi-Frimpong et al. (49)	Malawi	Cross-sectional study (two groups’ comparisons)	1000, ≥18 years	Community (household)	Sustainable farming techniques, such as organic composts; intercropping; mulching of stubble; and no-till farming. [I: agroecology-adoption (*n* = 571), C: non-agroecology-adaption (*n* = 429)]	NR	Health and well-being (self-reported), food insecurity access (Household Food Insecurity Access Scale-HFIAS)	Agroecological-conscious households were more likely to report ideal health status (OR = 1.37, *p* = 0.05), According to the average treatment effect, adopters had a 12% higher chance of being in optimal health. Moderate food insecurity (OR = 0.59, *p* = 0.05) and severe food insecurity (OR = 0.89, *p* = 0.10) were linked to a lower chance of reporting optimum health status.
**Nutritional interventions/Developed countries (poverty regions)**										
	7.	Ball et al. (46)	Catawba County, North Carolina (United States)	Intervention study (Pre-post survey)	415, NR	Community (farmers)	● Given Farmers’ Market Nutrition Program (FMNP) coupons weekly for 24 weeks to observe the marketing behaviors (I: 415 farmers).	72 months	fresh fruits and vegetables (F and V), quality of fresh F and V, and the ability to purchase food grown locally and increase the FMNP coupon redemption rate	Between 2007 and 2013, the average redemption rate was 51.4% (rank of 10). In 2013, the redemption index increased from 51.3 to 62.9% (rank of 3).
	8.	Breckwich et al. (44)	San Francisco, California (United States)	Intervention study (Pre-post survey)	Customers, NR	Community (stores)	● Advertising the products in the pilot store through promotional giveaways of canvas shopping bags, taste testing, fresh produce, and conducting in-store press events. ● Working with local graphic artists to develop and implement an award-winning media campaign to raise awareness about the program. ● Coverage of the pilot store intervention by several local media sources	6 months	To execute an inventive and sustainable public policy solution to expand community access to healthy food	In 3 years, this partnership achieved a remarkable number of policy-related successes, including youth-led research on the issue of food insecurity, a fruitful pilot Good Neighbor store intervention, community outreach, and education to influence community knowledge and behavior change, the development of a city- and foundation-sponsored initiative to expand the Good Neighbor Program, and state legislation to support similar initiatives across California.
	9.	Dailey et al. (45)	Adams County, Colorado, United States	Intervention study (Pre-post survey)	47 Latino or Hispanic (81.6% Hispanic), NR	Community (household)	● Given vouchers to families for increasing fruit and vegetable consumption	4 months	Food security (accessibility), fruit and vegetable intake, increased ability to purchase healthy, fresh foods	34.2% of participants reported consuming 2–3 servings of fruits and vegetables daily, and two-thirds of the respondents reported four fruits and vegetables per day. Over 40% of respondents reported greater stress related to having enough money to purchase nutritious meals.
	10.	Rollins et al. (47)	Atlanta, Georgia (United States)	Intervention study (Pre-post survey)	11 corner 100 African American corner store customers (≥18 years) with little access to nutritious foods	Community (corner stores)	● Stores sold fresh, healthy products and promoted cleanliness of store labeling the products. A customer intercept survey was administered at five healthy corner store locations to African American customers.	12 months	Assessing the Healthy shopping behavior	80% of customers said that they would purchase healthy food, e.g., fresh and inexpensive vegetable and fruits if sold in corner stores
Randomized controlled trials	11.	Jernigan et al. (53, 54)	Oklahoma, United States	cluster randomized controlled trial	1204, ≥18 years Chickasaw Nation (403 control, 410 intervention) and Choctaw Nation (409 control, 415 intervention)	Community (stores)	● Improve convenience stores and healthy retail strategies recommended by the Institute of Medicine and Centers for Disease Control, including (1) increasing availability, variety, and convenience of healthy foods; (2) placement of point of purchase details; (3) promoting, advertising, and selling nutritious foods; and (4) reducing healthy foods prices measured by NEMS tools to assess objective changes in the nutrition environment of the stores before and after the intervention. (I: two stores received the intervention and C: two stores did not receive the intervention)	9–12 months	Individual-level outcomes: ● Increases in fruit and vegetable consumption and secondary outcomes included consumption of other foods, changes in the perceived food environment, recall of promotions, and reported purchase of healthy foods. Store-level outcomes: ● Availability of healthy foods with an emphasis on ready-to-eat fruits and vegetables, in addition to pricing, placement, and quality measured by NEMS tools	Individual-level outcomes: After the intervention, both control and intervention participants’ daily fruit and vegetable intake stayed low. Following the intervention, both intervention and control participants believed that stores were healthier. Purchases of fruits, vegetables, and other healthy items were linked to higher shopping frequency. Store-level outcomes: There were no variations in the median NEMS scores between intervention and control stores.
**Nutritional interventions/Developing countries**										
	12.	Kang et al. (41)	Ethiopia	cluster randomized controlled trial	1790, 6–12 months	Community (mothers)	● Education of mothers in 12-day nutrition classes focused on child feeding that follows the ’learning by doing approach. I: (*n* = 876), C: (*n* = 914)	15 months	Weight-for-age (WAZ), weight-for-length/height z score (WLZ/WHZ), and Length/height-for-age z score (LAZ)	Children in the intervention area had faster growth in length [difference (diff): 0.059 cm/month; 95% CI: 0.027, 0.092; *p* = 0.001] and weight (diff: 0.031 kg/month; 95% CI: 0.019, 0.042; *p* < 0.001). The monthly changes in WAZ (diff: 0.028 z score/month; 95% CI: 0.016, 0.039) and WLZ (diff: 0.042 z score/month; 95% CI: 0.024, 0.059) were also found to be slower, favoring children in the intervention area, compared with the control area (all *Ps* < 0.001).

NR, non-reported; I, intervention; C, comparison.

None of the studies presented sample size calculations and formulas. Boedecker et al. (51) only calculated the minimum required sample size based on a proposed formula and chose the MDD for young children as the impact indicator in this formula.

The sample size in included studies ranged from 47 to 1790 participants, who were 6 months to 65 years of age. Three studies included children or households in which children lived (43, 51, 54). Of these, two studies included households with children under 2 years of age (51, 54). Seven studies included adults (41, 43, 46, 49–52), three included farmers (46, 50, 52), two included women 15–49 years (51), and mothers (41). Two studies included rural households and customers without specifying their ages (44, 48).

### Characteristics of agricultural interventions

Strategies used in the six studies reporting agricultural interventions included agricultural education of farmers (43, 49–52), preparing and improving soil and seeds (43, 52), promoting and supporting gardening/harvesting by honoring and utilizing traditional skills and local culture (51), community gardening (43), improving household income (48), and using agroecological and sustainable farming practices (49, 50, 52). Agroecological projects harnessed local resources and used a farmer-to-farmer knowledge-sharing approach to train smallholder farmers on applying agroecological practices to improve agricultural productivity and household nutrition. These agroecological projects have been conducted in poor and land-locked countries such as Malawi. Almost one-third of Malawian households experience severe food insecurity, and the roles of colonial and postcolonial policy failures and environmental change have been widely acknowledged as drivers of food insecurity in the Malawian context.

### Characteristics of nutritional interventions

Using strategies in nutritional interventions were store and shopping programs (44, 47, 53), Fresh Fruit and Vegetable programs (45, 46), nutrition education programs for mothers (41), farmers market nutrition programs (46), and voucher/food assistance program (41, 45, 46). For example, in the food assistance program, each participating family received 40 dollars in vouchers per month to spend at farmers’ markets (45).

### Implementation methods of the interventions

A wide variety of techniques were implemented in the interventions, including educational workshops (43, 47, 51), cooking sessions (51), farmer-to-farmer knowledge-sharing approaches (50), media campaign (44), providing fresh fruit and vegetable products (46), healthy retail strategies through taste-testing, a cooking program to provide quick packs of fruits and vegetables, offering healthy foods at discount prices, and promotional signage (53, 54), individual nutrition counseling (41), casing fruiting technique (48), improving purchasing environment through fresh stock produce other healthier food options, label and promote healthy items, and maintain store cleanliness and appearance (47), food vouchers (45), different agroecological practices, such as making organic compost, stubble mulching, and intercropping (49), seed distribution (52).

### Setting

All studies were conducted in community settings. Specifically, six (50%) of the research engagement was at the household level (43, 45, 48, 49, 51), four (33.3%) at the individual level (41, 46, 52), and one included both household and individual level (50). Three studies took place in the store setting (44, 47, 53).

### Outcomes

All agroecological practices achieved statistically significant results in the desired food security objective (s). However, nutritional interventions were ineffective for some access components such as mean adequacy ratio (51), fruit and vegetable intake, and nutrition environment of the stores (53, 54).

The main outcomes were improved in the following dimensions of food security:

• Availability: household production diversity (50), increasing crop diversity (52), redemption rate (46).
• Access: household dietary diversity (50), Dietary Diversity Score (DDS), Minimum Dietary Diversity (MDD) (51), accessibility to healthy food (53), ability to purchase fresh fruits and vegetables grown locally (46), assessment of the healthy shopping behavior (47), increasing gross domestic product per capita, plant-based protein intake, and income generation in local markets (48).
• Utilization: length/height-for-age z score (LAZ), weight-for-length/height z score (WLZ/WHZ), weight-for-age (WAZ) (41).
• Stability: no studies evaluated stability outcome components of food security.

Two of the study directly evaluated food security outcomes (49, 52) (Table 4).

**TABLE 4:** Summary of community partners of the interventions and their significant effect on the food security dimensions and their components by the quality level of included studies.

Quality	Study	Community partners	Dimensions and components affected by the interventions				
			**Food security**	**Availability**	**Access**	**Utilization**	**Stability**
	Ball et al. (46)	Farmers’ Market Nutrition Program (FMNP), Women, Infants and Children (WIC), Catawba County Public Health Farmers’ Market (CCPH FM), Eat Smart Move More, local farmers, UNCG	−	● The average redemption rate was 51.4% between 2007 and 2013 (rank of 10). The rate of redemption in 2013 increased from 51.3 to 62.9% (rank of 3)	−	−	−
Weak	Boedecker et al. (51)	Community health volunteers (CHVs)	−	−	● Children’s mean DDS (effects of treatment = 0.7, *p* < 0.001)Children reaching MDD (effects of treatment = 0.2, *p* < 0.001)	−	−
	Breckwich et al. (44)	Non-profit youth empowerment, Literacy for Environmental Justice (LEJ), environmental justice education organization, and Public Health’s Tobacco Free Project (TFP)	−	−	● Achieve an innovative and sustainable public policy strategy to increase community access to nutritious food	−	−
	Carney et al. (43)	Oregon Clinical and Translational Research Institute (OCTRI), Nuestra Comunidad Sana uses (the Community Health Worker model), Oregon Health and Science University (OHSU), National Institutes of Health National Center for Research Resources, community group staff		−	● Vegetable intake increased from 18.2 to 84.8% (*p* < 0.001) in adults ● Vegetable intake increased from 24.0 to 64.0% (*p* = 0.003) in children	−	−
	Dailey et al. (45)	Adams County Food Policy Council (ACFPC), Adams County Farmers’ Market, Community Supported Agriculture (CSA)		−	● 34.2% of participants reported consumption of 2–3 servings of fruits and vegetables daily ● Two-thirds of respondents reported four or more fruits and vegetables daily	−	−
	Kazige et al. (48)	Local farmers	−	● Increasing gross domestic product per capita [Maise gave the highest mushroom yields (2.4 kg kg^–1^)]	● 43.5% of the households used common beans for consumption as a vegetable ● 20.7% of the households sold common beans at the local market	−	−
	Madsen (52)	Soils, Food and Healthy Communities, non-profit organizations in Malawi, local enumerators, non-profit staff, translators, and researchers	The percentage of food security increased from 10% at baseline to 30% at endline	● Increasing crop diversity on intervention farms from a mean of 2.06 crops to 4.23 crops	−	−	−
	Rollins et al. (47)	Residents, academic institutions, and social service agencies			● 80% of customers said that they would purchase healthy food, e.g., fresh and inexpensive vegetable and fruits if sold in corner stores		
Moderate	Kang et al. (41)	Rural Eastern Ethiopian communities, female operators, Health Extension Workers, and community volunteers, 18 enumerators	−	−	−	Children in the intervention area had ● Faster growth in length [difference (diff): 0.059 cm/month; 95% CI: 0.027, 0.092; *p* = 0.001] and weight (diff: 0.031 kg/month; 95% CI: 0.019, 0.042; *p* < 0.001). ● Monthly changes in WAZ (diff: 0.028 z score/month; 95% CI: 0.016, 0.039) and WLZ (diff: 0.042 z score/month; 95% CI: 0.024, 0.059)	−
	Kansanga et al. (50)	Trained enumerators who were fluent in the local languages (Tumbuka and Chichewa)	−	● Positive significant effect on household production diversity (β = 0.289, *p* < 0.01)	● Positive significant effect in household mean dietary diversity (β = 0.175, *p* < 0.01)	−	−
	Jernigan et al. (53, 54)	Trained tribal collaborators, tribal-university	−	−	● Participants perceived healthier stores after the intervention ● Higher shopping frequency was related to purchases of fruits, vegetables, and healthy items	−	−
	Nyantakyi-Frimpong et al. (49)	Trained enumerators familiar with the local language	Food security improvement in agroecological adopting households			● Agroecological-adopting households have more optimal health compared with non-adopting households (adopters were 12% more likely to be in health status)	−

### Food security and its dimensions validated measures

Two out of twelve studies assessed food security by validating Household Food Insecurity Access Scale (HFIAS) (49, 52). In one study, validated tools were used to measure the availability of ready-to-eat fruit and vegetable products as healthy foods and to assess the perceived nutrition environment (53) (Table 4).

### Community partners

In four studies, locally trained enumerators completed the questionnaires in the local language (41, 49, 50, 52). Community partners in other studies included: *universities* [Oregon Health and Science University (OHSU) (43), tribal-university] (53), *institutes* [Oregon Clinical and Translational Research Institute (OCTRI), National Institutes of Health, National Center for Research Resources (43), academic institutions] (47), *markets* [Catawba County Public Health Farmers’ Market (CCPH FM)], Women, Infants, and Children (WIC) Farmers’ Market Nutrition Program (FMNP) (46), Adams County Farmers’ Market (45), *community health workers* [Community Health Volunteers (CHVs) (51)], Nuestra Comunidad Sana [the Community Health Worker model (43), Health Extension Workers (41)], *non-profit organization* [Soils, Food and Healthy Communities non-profit organization in Malawi (52), non-profit youth empowerment and environmental justice education organization (44)], *Adams County Food Policy Council (ACFPC*), *Community Supported Agriculture (CSA*) (45), *Literacy for Environmental Justice (LEJ), and Public Health’s Tobacco Free Project (TFP*) (44), non-profit staff, translators, and researchers (52), local farmers (46, 48), community group staff (43), trained tribal collaborators (53), rural Eastern Ethiopian communities, female operators, community volunteers (41), Eat Smart Move More (46), residents, and social service agencies (47).

Of 12 studies, more than 80% (10 in all) reported community participation in the intervention design and implementation. Local farmers, program administrators, and community advocates were involved in setting priority, generating hypotheses, and documenting the implementation process. In two studies, local community cooperatives had only participated in data collection (49, 50). Institutes aided the communities and academic partners attain research funding, and researchers supervised and led the research process.

In two studies (16.66%) (44, 45), communities and non-profit organizations were involved in translating research findings into policy-changing, managing, and sustaining the program or interventions (Table 4).

### Formative research

Ten studies (83.3%) employed formative research to guide intervention development and implementation. Most of these (90%) achieved statistically significant results in the desired food security objective(s). Information on community needs, perceptions, and values was collected primarily during the planning phases before the intervention began, often through inputs obtained from community partners or stakeholders. Some studies also included formative assessments during or after the intervention to evaluate program suitability and participant satisfaction. Different data collection methods were identified as follows: interviews in four studies (46, 48, 49, 51), focus groups in two studies (44, 53), and qualitative or quantitative surveys in four studies (43, 45, 47, 52). Less commonly, direct observation was also used as another formative research approach (46).

Although none of the studies mentioned the challenges of implementing the interventions, some of the studies treated the possible challenges in implementing community-based participatory interventions with formative research. For example, Rollins et al., through spatial analysis and environmental assessment, evaluated the corner stores to confirm their existence, location, food offerings, and how residents use them and then developed a CBP intervention that was matured and appropriately matched and tailored to the community needs and environment.

### Methodology quality

The quality of all eligible studies (*n* = 12) was rated as moderate/weak, among which most of the studies were weak (*n* = 9, 75%). Information on study selection, confounders, blinding, and withdrawals were under-reported in most studies. Due to the nature of the intervention, it was assumed that no blinding was applied in some studies, and therefore, they were included in the category of moderate-quality studies. Selection bias, confounders, withdrawals, and blinding were considered the most common methodological problems among the underpowered studies. The results mainly came from studies with no report adjustment for confounders, which led to the weak global rating for most studies (*n* = 7, 58.33%) based on the EPHPP assessment. Among these, only three studies (25%) have adjusted with confounding factors such as education level, family size, household wealth, and farm size.

**FIGURE 2:**
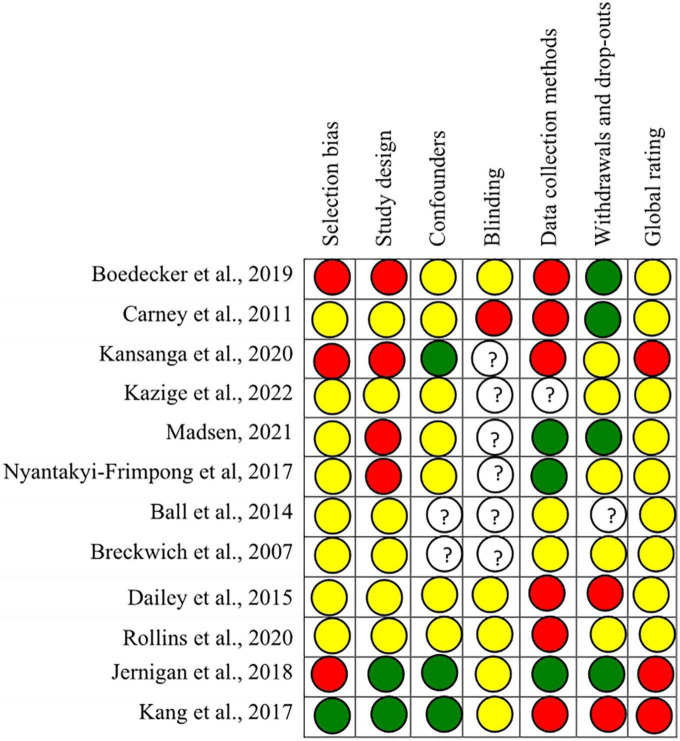
Quality assessment (using the EPHPP) of included studies (*n* = 12). 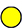 W, weak; 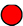 M, moderate; 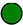 S, strong.

Of the twelve included studies, nine studies (75%) failed to provide reliability and validity of data collection tools (Figure 2).

## Discussion

There is widespread acknowledgment and appreciation of the important role that community-based participatory interventions play in addressing diverse health issues in developing countries (55–57). However, there is a little known about their impact on food insecurity. It seems that this systematic review is the first to examine community-based participatory interventions to improve food security.

Based on the CBPR conceptual framework models (14, 58), in all included studies, the CBPR started with participatory identification of an issue and progressed toward formulating research questions, developing intervention strategies and activities, implementing and implementing, and finally evaluating the impact of interventions.

A wide variety type of community-based participatory interventions was found, which we categorized under two main groups agricultural and nutritional categories were implemented. Agricultural workshops, preparing and improving soil and seeds, and agroecological practices as the most popular community-based participatory agricultural interventions were promising strategies to reduce food insecurity in developing countries. This study confirms the hypothesis that a more comprehensive adoption of agroecological approaches enhances the chance of food security improvement (59, 60). It was identified that when crop diversification is the main agroecological practice, diets were improved through the consumption of a greater variety of available foods. Furthermore, agroecological practices such as organic soil amendments, intercropping and botanical pesticides could reduce costs, saving income for purchasing food (61, 62).

In our review, the most commonly studied agroecological interventions, including crop diversification and soil management, compost/manure application, crop rotation, mulching, and agroforestry, were positively associated with more product diversity, improved yield and more production stability and so had increased income. Positive impacts of agroecological practices on food security and nutrition outcomes were documented in those studies that included multiple components (e.g., farmer-to-farmer knowledge-sharing approach, mixed crop-livestock systems, and crop diversification) (61).

Participatory agroecological interventions encompass social and cultural aspects of whole food systems and are based on localness, participation, fairness, and justice, which are important principles of food security and nutrition (63). The agroecological approach addresses resource use efficiency through practices that reduce or eliminate the use of costly, scarce, or environmentally damaging inputs. These practices and social movements are leading to transitioning agricultural and food systems to achieving global food and nutrition security and building a sustainable food system.

Another interesting finding of this review is that certain regions seem to focus on a specific domain of interventions. Developing countries were the most common regional target for agricultural interventions. However, developed countries, mainly Latin American countries, were the main target of the nutritional interventions. One possible explanation is more prevalence of inappropriate agriculture practices in low-income countries. Evidence confirms that agricultural programs contribute to improving nutritional status and food security in developing countries and poor areas (64–67). Agricultural interventions, especially agroecological practices, can be considered an alternative method to capitalize on community capacity to implement acceptable methods and promise to reduce food insecurity in developing countries (59, 60).

The findings of this study indicate a lack of consensus on best research or intervention practices due to wide variation in intervention efficacy and insufficient study quality to allow the generalization of findings. However, it could be argued that formative research data were more likely to increase the effectiveness of community-based food security programs. Formative research had significantly contributed to formulating and modifying culturally relative interventions and optimizing intended effects, which was consistent with other review studies (26). Within the CBPR framework, formative research is often employed to culturally tailor interventions to the population of interest, especially when targeting food security and health promotion outcomes (68–70) to inform and execute interventions that take into account community attitudes, needs, and barriers (71).

A key finding of this review was insufficient data on the stability components of food security, and weak study designs were the considerable gaps in the research evidence reviewed. Since no high-quality studies were found, comparing the findings of high- and low-quality studies was impossible. However, it seems that the difference in the effectiveness of the results is more due to the type of interventions than their quality. The stability of prices and supplies is a crucial dimension in food security, which improves households’ capacity to respond and adapt to shocks. Regarding the importance of agriculture to the economies of rural areas both in developed and developing countries, this sector can contribute to improving food stability, as well as to furthering food security (42), which was not addressed in the studies reviewed.

Furthermore, no validated tools used in the reviewed studies addressed all food security dimensions. The development of precise tools for measuring food insecurity and its dimensions and adopting a unified approach will provide a foundation for developing effective programs to improve food security (72).

## Strengths and limitations of the study

the application of a comprehensive and sensitive search strategy through four databases to identify all potentially relevant peer-reviewed papers and gray literature could be considered the main strength of this review. Using independent reviewers throughout the review process also improved the quality of the methodology.

However, despite the rigorous and novel approach, our review has some noteworthy limitations. This study included only peer-reviewed journal articles, so there is a chance of missing those published as organizational reports and documents (publication bias). Furthermore, it reviewed only English papers focused on household and community-based studies, so there is a chance of missing some useful non-English papers reporting interventions conducted in other settings. However, in order to increase the sensitivity of the search, setting-related keywords were not included in the search strategy, and we did not find any community-based participatory interventions conducted in clinical settings. The total sample size of the studies included in the review was less than 10,000, which makes it difficult to draw definitive conclusions. The low to moderate quality of all reviewed studies was another limitation that limited the possibility of comparing the effectiveness of participatory interventions based on their quality. Finally, a meta-analysis of the effect size of interventions was not possible due to heterogeneous study designs and outcome measures; therefore, a descriptive analysis was performed.

## Conclusion

This review emphasizes the value of community-based participatory programs to tackle food insecurity, highlighting community-based food security improvement strategies and a vast list of techniques and methods. Most programs adopted a community-based participatory approach in the intervention design and implementation. Local farmers, program administrators, and community advocates were involved in setting priority, generating hypotheses, and documenting the implementation process. It was found that CBP interventions guided by formative research data and agroecological practices were promising strategies for improving food security and its dimensions. Agroecological projects harnessed local resources and used a farmer-to-farmer knowledge-sharing approach to train smallholder farmers on applying agroecological practices to enhance agricultural productivity and household nutrition (availability and access dimensions). However, nutritional interventions effectively improved access, availability, and food utilization. No studies evaluated stability outcome components of food security.

## Suggestions for future research

Insufficient data on the stability components of food security and weak study designs were the considerable gaps in the research evidence reviewed. The scarcity of addressing the social, demographic, political, economic, and environmental variability in the reviewed studies as influencing factors of food security and/or its dimensions requires improvement in future research. Hence, future research should pay more attention to the stability components of food security and the quality of methodology. More randomized experimental research with large sample sizes and high retention rates is needed to strengthen the evidence on best CBPR practices to improve food security.

## Data availability statement

The original contributions presented in this study are included in the article/Supplementary material, further inquiries can be directed to the corresponding author/s.

## Author contributions

NK-M, FM-N, and AD conceived and designed the study. AD, FM-N, SA, and MH-S developed the search strategy. AD and MH extracted the data. All authors contributed to developing and finalizing the manuscript.

## Footnotes

1
https://ethics.research.ac.ir/ProposalCertificateEn.php?id=166308&Print=true&NoPrintHeader=true&NoPrintFooter=true&NoPrintPageBorder=true&LetterPrint=true

2
www.worldbank.org
