# Supplementary material for: Community-based participatory interventions to improve food security: A systematic review
Source: Front Nutr. 2022 Dec 19;9:1028394. doi: 10.3389/fnut.2022.1028394 (PMC9807164; doi:10.3389/fnut.2022.1028394)
Supplement: Supplementary file 1 [file Table_1.DOCX]

**Table S1. PICO^1^ and database search strategy through February 2022**

| **PICO components** | | |
| --- | --- | --- |
| **Population** | children, adolescents, adults, or older adults and socio-economically disadvantaged groups | |
| **Intervention** | community-based participatory (CBP) interventions to improve food security indicators | |
| **Comparison** | All comparisons: different educational intervention; various methods of delivery, educational contents, intervention dosages, or the like; usual care; with or without control groups | |
| **Outcome** | primary outcomes: dimensions and components of food and nutrition security, including food availability, accessibility, utilization, and stability  secondary outcomes: land used for agriculture, the density of supermarkets or retail outlets selling nutritious foods, or adverse outcomes, including stigmatization, dependency, obesity, or excessive weight loss | |
| **Searches in Scopus, PubMed, Web of science, and EMBASE** | | |
| **Database** | **Search terms** | **Item found** |
| **Scopus search strategy** | | |
| **#1** | TITLE ( Food* OR nut* OR nourish* OR eat* OR diet* OR feed* OR malnut*) | 1,151,826 |
| **#2** | TITLE ( Secur* OR insecur* OR supply OR reserve* OR insuff* OR affluen* OR suffice* OR avail* OR access* OR stabil* OR utilize*) | 1,400,988 |
| **#3** | TITLE ( commun* OR participat* OR cbpr OR action OR collaborat* OR empower* OR polic* OR program* OR intervene*) | 2,138,253 |
| **#4** | #1 AND #2 AND #3 | **2329** |
| **PubMed search strategy** | | |
| **#1** | food*[Title] OR nutr*[Title] OR nourish*[Title] OR diet*[Title] OR feed*[Title] OR malnutr*[Title] OR "food" [MeSH Terms] OR "diet" [MeSH Terms] | 1,214,629 |
| **#2** | secur*[Title] OR insecur*[Title] OR supply [Title] OR reserve*[Title] OR insuff*[Title] OR affluen*[Title] OR suffice*[Title] OR avail*[Title] OR access*[Title] OR stabil*[Title] OR utilize*[Title] OR "food security" [MeSH Terms] OR "food insecurity" [MeSH Terms] OR " food supply " [MeSH Terms] | 426,905 |
| **#3** | commun* [Title] OR participat* [Title] OR cbpr [Title] OR action [Title] OR collaborat* [Title] OR empower*[Title] OR polic*[Title] OR program*[Title] OR intervene*[Title] OR "community-based participatory research"[MeSH Terms] | 792,952 |
| **#4** | #1 AND #2 AND #3 | 2,146 |
| **Web of science search strategy** | | |
| **#1** | TITLE: (Food* OR nut* OR nourish* OR eat* OR diet* OR feed* OR malnut*) | 1,144,731 |
| **#2** | TITLE: (Secur* OR insecur* OR supply OR reserve* OR insuff* OR affluen* OR suffice* OR avail* OR access* OR stabil* OR utilize*) | 1,097,727 |
| **#3** | TITLE: (commun* OR participat* OR cbpr OR action OR collaborat* OR empower* OR polic* OR program* OR intervene*) | 1,985,273 |
| **#4** | #1 AND #2 AND #3 | **2193** |
| **EMBASE search strategy** | | |
| **#1** | food*:ti OR nut*:ti OR nourish*:ti OR eat*:ti OR diet*:ti OR feed*:ti OR malnut*:ti | 676,869 |
| **#2** | secur*:ti OR insecur*:ti OR supply:ti OR reserve*:ti OR insuff*:ti OR affluen*:ti OR suffice*:ti OR avail*:ti OR access*:ti OR stabil*:ti OR utilize*:ti | 522,416 |
| **#3** | commun*:ti OR participat*:ti OR cbpr:ti OR action:ti OR collaborat*:ti OR empower*:ti OR polic*:ti OR program*:ti OR intervene*:ti | 862,849 |
| **#4** | #1 AND #2 AND #3 | **932** |

^1^ Due to the increased sensitivity of the search, the setting/study design was not included in the search strategy
