# Supplementary material for: Community-based participatory interventions to improve food security: A systematic review
Source: Front Nutr. 2022 Dec 19;9:1028394. doi: 10.3389/fnut.2022.1028394 (PMC9807164; doi:10.3389/fnut.2022.1028394)
Supplement: Supplementary file 2 [file Table_2.DOCX]

Table 1. Excluded studies

| **Study** | **Reason for exclusion** |
| --- | --- |
| Nyantakyi-Frimpong et al, 2016 ([1](#_ENREF_1)) | Irrelevent study design (no intervention study), the study records a qualitative methology. |
| Sparkle Springfield et al, 2016 ([2](#_ENREF_2)) | Intervention aims at improving the anthropometrics, diet and physical activity (irrelevent study outcome). |
| Jarrott et al, 2019 ([3](#_ENREF_3)) | Irrelevent study design (no intervention study). The study is related to develop a community-based participatory intervntion to address food insecurity, and there is no implementation and evaluation of the study. |
| Marina Villegas et al, 2020 ([4](#_ENREF_4)) | Irrelevent study design (no intervention study), the study records a qualitative methology. |
| Isabel C. Scarinci et al, 2014 ([5](#_ENREF_5)) | Irrelevent study outcome (no information on food security). The study records healthy lifestyle intervention to decrease in fried food consumption and improve fruit/vegetable intake and physical activity among women to prevent the breast and cervical cancer. |
| Kazige et al, 2022 ([6](#_ENREF_6)) | Irrelevent study design (no intervention study) |
| Beck et al, 2014 ([7](#_ENREF_7)) | Wrong approche and setting. The study is a clinical-community intervention (in clinical-care setting) instead of community-based participatory research to address food insecurity |

**References**

1. Nyantakyi-Frimpong H, Mambulu FN, Kerr RB, Luginaah I, Lupafya E. Agroecology and sustainable food systems: Participatory research to improve food security among HIV-affected households in northern Malawi. Social Science & Medicine. 2016;164:89-99.

2. Springfield S, Buscemi J, Fitzgibbon ML, Stolley MR, Zenk SN, Schiffer L, et al. A randomized pilot study of a community-based weight loss intervention for African-American women: Rationale and study design of Doing Me! Sisters Standing Together for a Healthy Mind and Body. Contemporary clinical trials. 2015;43:200-8.

3. Jarrott SE, Cao Q, Dabelko-Schoeny HI, Kaiser ML. Developing intergenerational interventions to address food insecurity among pre-school children: A community-based participatory approach. Journal of Hunger & Environmental Nutrition. 2021;16(2):196-212.

4. Villegas M. Planty for the People: A Community-Based Participatory Research Approach to Healthy, Plant-Based Food Access in the Border Region: The University of Texas at El Paso; 2020.

5. Scarinci IC, Moore A, Wynn-Wallace T, Cherrington A, Fouad M, Li Y. A community-based, culturally relevant intervention to promote healthy eating and physical activity among middle-aged African American women in rural Alabama: findings from a group randomized controlled trial. Preventive medicine. 2014;69:13-20.

6. Kazige OK, Chuma GB, Lusambya AS, Mondo JM, Balezi AZ, Mapatano S, et al. Valorizing staple crop residues through mushroom production to improve food security in eastern Democratic Republic of Congo. Journal of Agriculture and Food Research. 2022;8:100285.

7. Beck AF, Henize AW, Kahn RS, Reiber KL, Young JJ, Klein MD. Forging a pediatric primary care-community partnership to support food-insecure families. Pediatrics. 2014;134(2):e564-71.
